# Cutaneous Presentation Mimicking Tuberculosis as an Initial Indicator of Hairy Cell Leukemia: A Case Report

**DOI:** 10.7759/cureus.76606

**Published:** 2024-12-30

**Authors:** Eduardo Macedo, Catarina Ferreira, Ana S Oliveira, Ana Rita Marques

**Affiliations:** 1 Internal Medicine, Hospital de Braga, Braga, PRT; 2 Oncology, Hospital de Braga, Braga, PRT

**Keywords:** cytopenia, flow cytometery, hairy cell leukemia, hematology, splenomegaly, tuberculosis

## Abstract

Hairy cell leukemia (HCL) is a rare and slow-progressing lymphoid disorder commonly presenting with splenomegaly and cytopenias. The diagnosis can be challenging due to its nonspecific clinical presentation, frequently resembling other diseases. We report the case of a 48-year-old male patient, whose initial diagnostic hypotheses included cutaneous tuberculosis and reactive arthritis, but the diagnosis was confirmed as HCL after further investigation, including flow cytometry. Treatment with purine analogs led to remission and improved the patient’s outcome. This case highlights the complexity of diagnosing HCL, particularly in atypical presentations, and emphasizes the importance of a multidisciplinary approach involving clinical, laboratory, and advanced diagnostic techniques. Despite its rarity, HCL should be considered in patients with unexplained cytopenias and splenomegaly.

## Introduction

Hairy cell leukemia (HCL), also referred to as leukemic reticuloendotheliosis, is an indolent and rare lymphoid malignancy defined by the growth of neoplastic B cells with large cytoplasm and hair-like extensions within the bone marrow, splenic red pulp and peripheral blood [1,2]. It is estimated that HCL accounts for approximately 2% of lymphocytic leukemia cases and is more prevalent in middle-aged men [1]. HCL usually causes variably reduced production of platelets, white blood cells, and red blood cells as well as splenomegaly. The cytopenias lead to systemic consequences, including an increased risk of infection, anemia, and bleeding [1-6].

Cutaneous tuberculosis, although it can occur at any age and in any sex, is more common in men and in individuals with compromised immune systems, such as in the case of hairy cell leukemia [7].

We present a case of a 48-year-old man with an unusual presentation of HCL, who presented to the Internal Medicine consult due to asthenia, frequent mouth ulcers, arthritis, and erythematous lesions. These symptoms ultimately led to the diagnosis of HCL, with initial suspicion of cutaneous tuberculosis.

## Case presentation

A 48-year-old man, a construction worker, ex-smoker (30 pack-year), and with moderate alcohol consumption (50 ml per day), presented with asthenia, frequent mouth ulcers, and arthritis in the large joints, with a migratory pattern associated with erythematous lesions on the lower limbs, resembling cutaneous tuberculosis lesions. He also had a history of nighttime sweating without fever or weight loss. These symptoms had been present for one month, and he had previously been medicated with prednisolone 60 mg per day due to an exuberant erythematous lesion and arthritis on the right lower limb. Upon physical examination, splenomegaly was noted, with the spleen palpable 3 cm below the left costal arch. Cutaneous tuberculosis and reactive arthritis were suspected, and the patient was followed up for further investigation.

**Figure 1:**
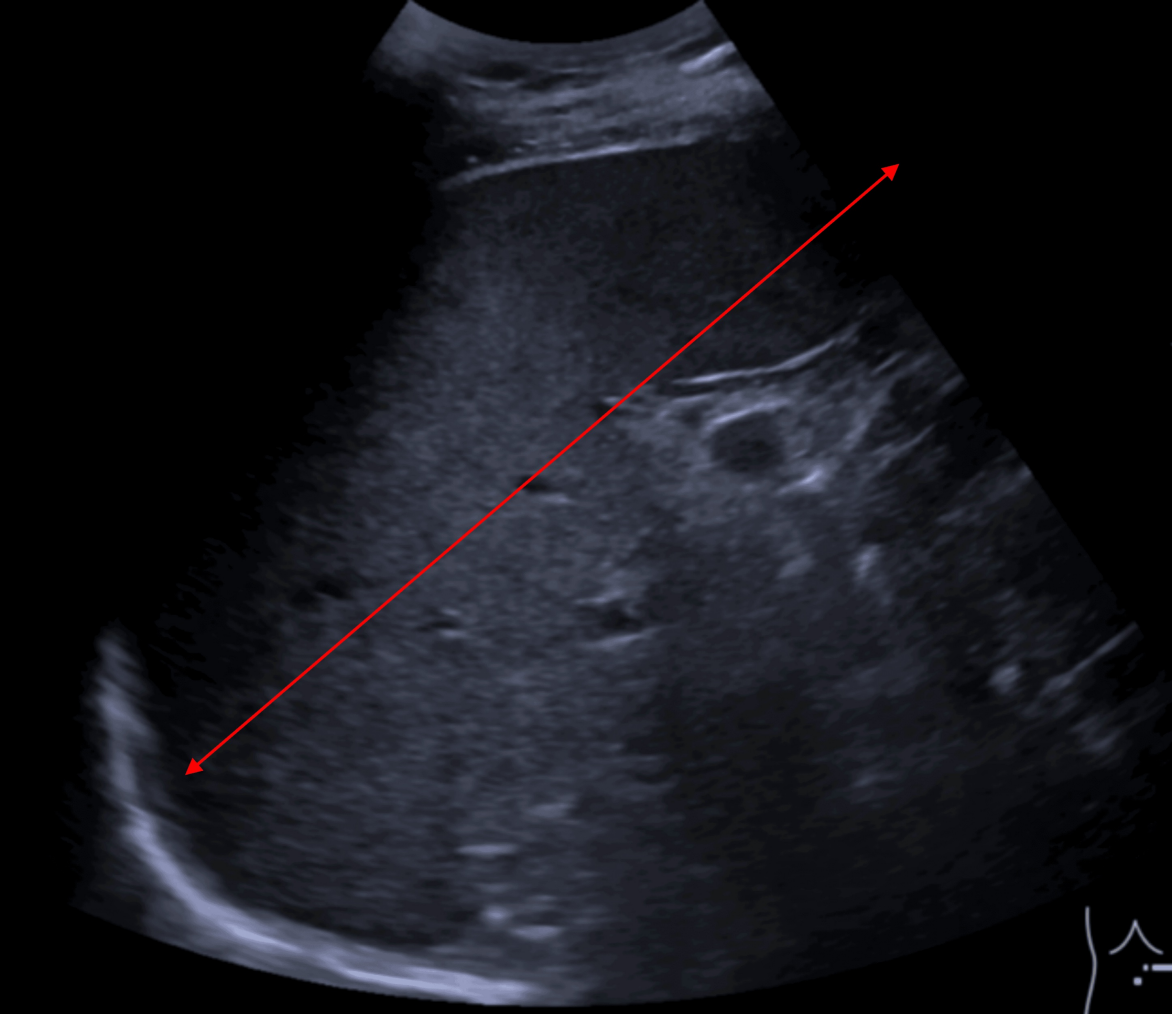
Echography showing splenomegaly with 17 cm bipolar axis (marked by the red arrow).

Bloodwork revealed thrombocytopenia, with a platelet count of 47,000/µL (normal range: 150,000-450,000/µL), and monocytopenia at 1% (reference range: 2-10%). The infectious study showed a positive interferon-gamma release assay (IGRA), while the autoimmune workup (antinuclear antibodies, rheumatoid factor, anti-cyclic citrullinated peptide (anti-CCP), human leukocyte antigen (HLA)-B27, complement levels, erythrocyte sedimentation rate, and protein electrophoresis) was negative. Echography confirmed splenomegaly, with a 17 cm bipolar axis (Figure 1).

**Figure 2:**
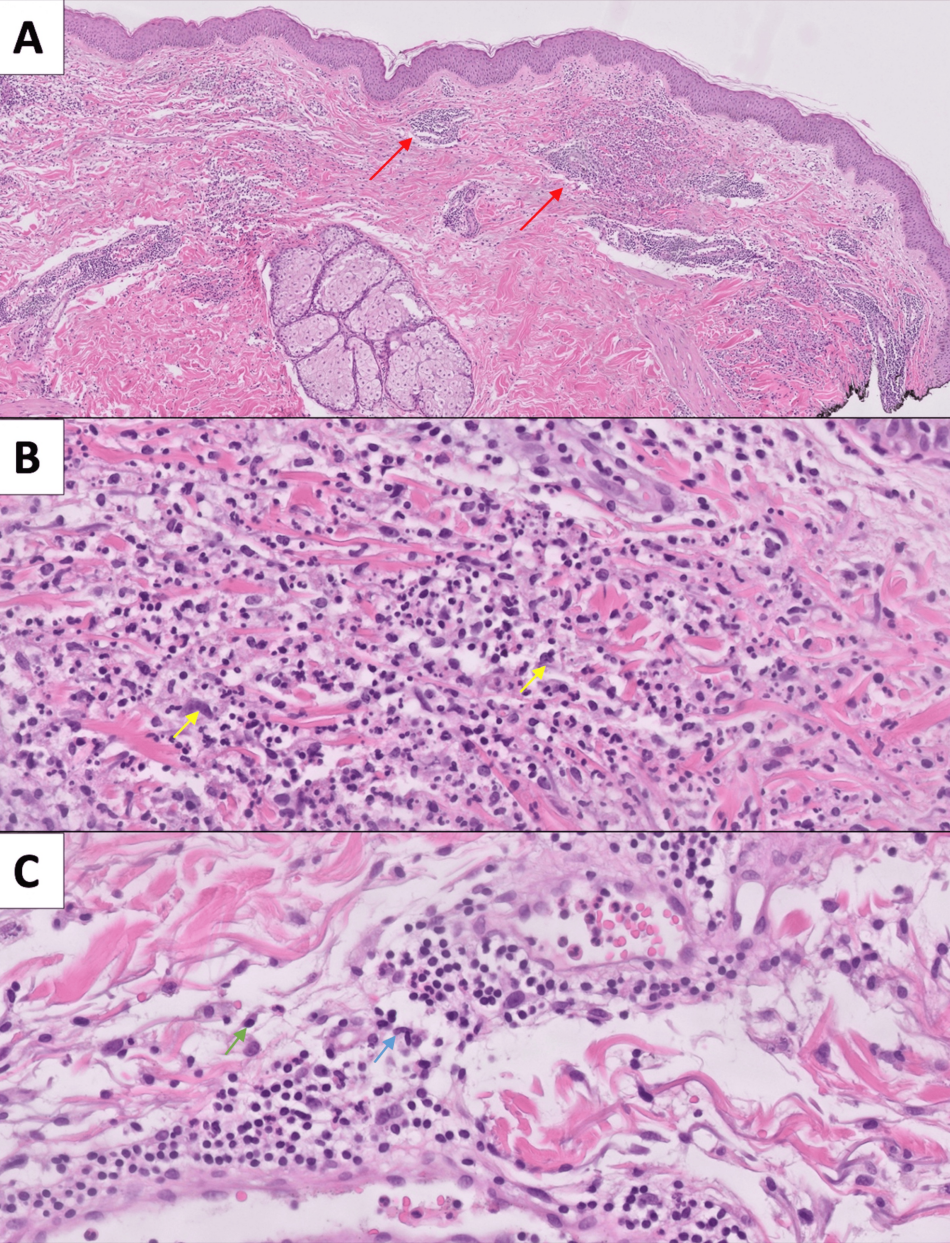
Histopathological examination of cutaneous biopsy (A) H&E 50X, Cutaneous biopsy showing a moderate inflammatory infiltrate localized in the superficial and mid-dermis (marked by red arrows), with interstitial and perivascular components; (B) H&E 400x, The interstitial inflammatory component is primarily neutrophilic, associated with abundant karyorrhexis (marked with yellow arrows); (C) H&E 400x, The perivascular component contains lymphocytes (marked with green arrow) and neutrophils (marked with blue arrow), with no evidence of vascular damage. Image Courtesy: Laboratório UNILABS, Braga

The biopsy later suggested neutrophilic dermatosis/Sweet's syndrome and did not show features indicative of cutaneous tuberculosis (Figure 2).

**Figure 3:**
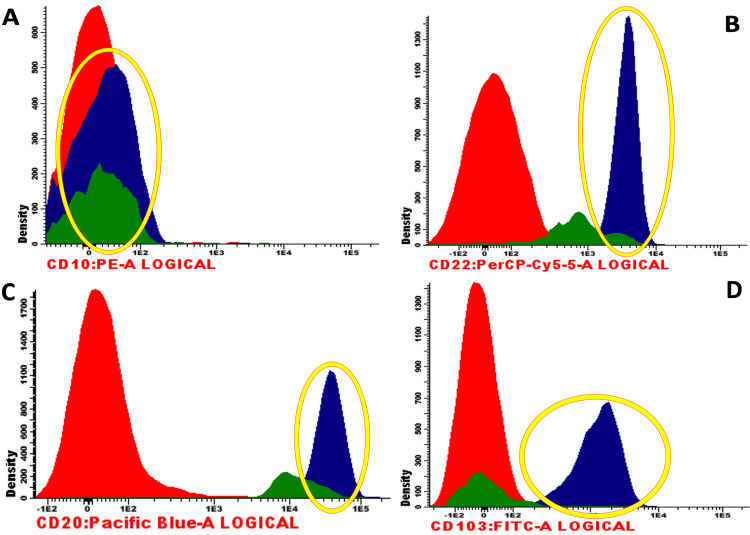
Flow cytometry analysis revealing an immunophenotypic profile of Hairy Cell Leukemia. (A) B-cell markers negative for CD10; (B) B-cell markers positive for CD22; (C) B-cell markers positive for CD20; (D) B-cell markers positive for CD103. Blue: Hairy Cell Leukemia B Cells (surrounded by a yellow circle); Green: Normal B Cells; Red: T Cells. Image Courtesy: Centro de Medicina Laboratorial Germano de Sousa

However, as the patient continued to have thrombocytopenia (60,000 platelets/µL) and monocytopenia (1%), flow cytometry was performed, revealing large-sized B cells and lymphocytes expressing CD19, CD20, CD22, CD200, and CD103 and no expression of CD5 and CD10 (Figure 3).

HCL was diagnosed, and the patient was referred to a Hematology consult. He began treatment with cladribine, resulting in hematological recovery and resolution of splenomegaly, and continued follow-up with periodic complete blood count tests and leukogram.

## Discussion

HCL is an uncommon, slow-progressing B-cell lymphoproliferative condition that primarily affects the reticuloendothelial system and frequently leads to bone marrow suppression and splenomegaly [1,2]. It accounts for about 2% of all leukemia cases [1]. Typical laboratory findings include thrombocytopenia and monocytopenia, and the resulting cytopenias can cause anemia, bleeding and an increased vulnerability to infections [1-4].

The pathogenesis of HCL remains incompletely understood [1]. Most cases are thought to arise from a late-stage activated memory B cell bearing the *BRAF* V600E gene mutation [3]. Although exposure to pesticides, farming activities, and ionizing radiation have been cited as possible risk factors, there is no strong evidence linking cigarette smoking, alcohol consumption, solvents, or obesity to an increased risk for HCL [1].

The clinical presentation of HCL is often indolent, with nonspecific symptoms such as fatigue, unintentional weight loss, and low-grade fever. Splenomegaly is a hallmark finding, present in most patients, frequently accompanied by pancytopenia, including neutropenia and thrombocytopenia, due to bone marrow infiltration and splenic sequestration. Patients may also experience recurrent infections or bleeding complications [1-6].

Diagnosis relies on a combination of clinical, morphological, and immunophenotypic/flow cytometry findings. Peripheral blood smear and bone marrow biopsy reveal the presence of hairy cells, which express markers such as CD11c, CD25, CD103, and annexin A1 and do not express CD5 or CD10 [6]. Also, *BRAF* V600E mutation can aid in diagnosing HCL. Treatment often involves purine analogs, such as cladribine, and remission has high success rates [8-11], as was the case with our patient.

## Conclusions

This case highlights the complexity and diagnostic challenges associated with HCL, particularly when atypical manifestations such as cutaneous lesions are the initial presentation. A definitive diagnosis requires a multidisciplinary approach that combines clinical data, laboratory tests, and advanced techniques, such as immunophenotyping and flow cytometry. Although the disease is rare, the availability of effective therapies, such as purine analogs, facilitates high remission rates and significantly enhances patient outcomes. This underscores the importance of maintaining a high index of suspicion, even when the clinical presentation is atypical.
